# Predicting Egg Storage Time with a Portable Near-Infrared Instrument: Effects of Temperature and Production System

**DOI:** 10.3390/foods13020212

**Published:** 2024-01-09

**Authors:** Daniel Cozzolino, Pooja Sanal, Jana Schreuder, Paul James Williams, Elham Assadi Soumeh, Milou Helene Dekkers, Molly Anderson, Sheree Boisen, Louwrens Christiaan Hoffman

**Affiliations:** 1 Centre for Nutrition and Food Sciences, Queensland Alliance for Agriculture and Food Innovation (QAAFI), The University of Queensland, St. Lucia, Brisbane, QLD 4072, Australia; louwrens.hoffman@uq.edu.au; 2 School of Agriculture and Food Sustainability, The University of Queensland, St. Lucia, Brisbane, QLD 4072, Australia; poojasanal@uq.net.au (P.S.); e.soumeh@uq.edu.au (E.A.S.); 3 Food Science Department, Stellenbosch University, Private Bag X1, Matieland, Stellenbosch 7602, South Africa; jana.schreuder@gmail.com (J.S.); pauljw@sun.ac.za (P.J.W.); 4 Queensland Animal Science Precinct (QASP), The University of Queensland, Gatton Campus, St. Lucia, Brisbane, QLD 4072, Australia; m.dekkers@uq.edu.au (M.H.D.); molly.anderson@uq.edu.au (M.A.); s.barnwell@uq.edu.au (S.B.)

**Keywords:** egg freshness, near-infrared spectroscopy, chemometrics, storage time, cage, free range

## Abstract

Determining egg freshness is critical for ensuring food safety and security and as such, different methods have been evaluated and implemented to accurately measure and predict it. In this study, a portable near-infrared (NIR) instrument combined with chemometrics was used to monitor and predict the storage time of eggs under two storage conditions—room temperature (RT) and cold (CT) storage—from two production systems: cage and free-range. A total of 700 egg samples were analyzed, using principal component analysis (PCA) and partial least squares (PLS) regression to analyze the NIR spectra. The PCA score plot did not show any clear separation between egg samples from the two production systems; however, some egg samples were grouped according to storage conditions. The cross-validation statistics for predicting storage time were as follows: for cage and RT eggs, the coefficient of determination in cross validation (R^2^_CV_) was 0.67, with a standard error in cross-validation (SECV) of 7.64 days and residual predictive deviation (RPD) of 1.8; for CT cage eggs, R^2^_CV_ of 0.84, SECV of 5.38 days and RPD of 3.2; for CT free-range eggs, R^2^_CV_ of 0.83, SECV of 5.52 days and RPD of 3.2; and for RT free-range eggs, R^2^_CV_ of 0.82, SECV of 5.61 days, and RPD of 3.0. This study demonstrated that NIR spectroscopy can predict storage time non-destructively in intact egg samples. Even though the results of the present study are promising, further research is still needed to further extend these results to other production systems, as well as to explore the potential of this technique to predict other egg quality parameters associated with freshness.

## 1. Introduction

Eggs are considered one of the least expensive sources of animal protein for humans, with an estimated annual consumption of 10.9 kg eggs/capita (OECD-FAO, 2022). Not only are eggs a rich source of protein, but they also provide omega 3 fatty acids, vitamins (e.g., Vitamins D, E, B5 and B12) and minerals (e.g., calcium, selenium) [1,2,3].

Egg freshness is one of the main factors that contributes to the overall quality of eggs and egg-derived products [1,2,3]. Freshness is one of the main parameters of the egg industry, with egg freshness critical for consumers who view it as a top-quality factor when buying this dietary staple [4,5]. This parameter also plays an important role in assuring the integrity of the supply chain of this commodity (e.g., transport and storage) [5,6]. A decrease in egg freshness is associated with changes in the biochemical and chemical composition within the egg. These include interactions between ovomucin and lysozyme, alterations in ovomucin disulfide bonds and changes in the carbohydrate moieties of the ovomucin, which are mainly involved in egg white thinning [7].

Several methods are used by the industry to measure egg freshness [6,8]. They include the measurement of egg weight, albumin height, protein content, eggshell strength, Haugh unit (HU) [9], egg shape index, eggshell color, air chamber height and egg-specific gravity [6,8]. The Haugh unit is the gold standard used by the egg industry to measure freshness, followed by the yolk index [5,6,8]. However, these two parameters only reflect a minimal percentage of the freshness quality of eggs, as they hardly integrate with the biochemical and chemical changes that are involved in explaining egg freshness or quality [5,6,8]. Recently, researchers have suggested that the determination f protein content can be included as a quality indicator for freshness [10], whereas other available methods include the measurement of albumen pH, air cell height, yolk index, and specific gravity [8]. However, these methods require the destruction of the egg during an analysis where a small number of samples are utilized to estimate freshness from a larger batch of samples [5,6,8].

Organic and free-range production systems have increased in many countries due to consumer preferences, as well as the increasing demand for high-quality foods, produced under more sustainable and improved welfare conditions [2,3,5]. Cage eggs are defined as those produced from hens that are housed in cages inside large, climate-controlled sheds. In Australia, the Model Code of Practice for the Welfare of Animals stipulates that the minimum space allowance per hen in a cage farming system is 550 cm^2^ per bird with a minimum cage height of 40 cm [2]. Cage hens are also referred to as battery hens as the parallel rows inside the sheds look like rows of cells inside a battery [2]. On the other hand, free-range eggs are considered as those produced from hens that have access to an outdoor range during the day but are housed securely and comfortably in sheds. To be classified as a free-range egg, the hens must have meaningful and regular access to an outdoor range during daylight hours [2]. Usually, consumers are willing to pay more for free-range eggs than cage eggs [2,5]. However, it is still challenging to identify and trace the provenance or the systems of production of eggs from unknown practices [5]. Research has also indicated that the system of production can affect the freshness of eggs [4,5].

Different, non-destructive methods have been evaluated and suggested by various researchers to measure and monitor egg freshness, including the utilization of electronic noses, the determination of dielectric properties and the use of machine vision technology [5,6] and vibrational spectroscopy such as near-infrared and hyperspectral imaging [5,8,11]. These methods have several advantages over the traditional ones, such as the non-destructive nature of the technology, the speed of the analyses and the low cost of the analyses [5,8,11].

Vibrational spectroscopy methods have been explored by various researchers to measure egg freshness [12,13,14]. Kemps and collaborators (2007) reported the use of both visible (VIS) and near-infrared (NIR) transmission spectroscopy combined with low-resolution proton nuclear magnetic resonance to assess egg HU [15]. Hyperspectral imaging has also been evaluated to predict HU and other parameters associated with freshness [16,17,18,19], bubble formation and scattered yolk [16]. The evaluation of different algorithms combined with NIR spectroscopy such as support vector data description (SVDD) [7], variable selection methods including spectral interval selection, genetic algorithm (GA) and successive projections algorithm (SPA) [20], and the use of the maximum likelihood method [21] have also been explored to predict egg freshness. Most of these studies predicted HU as an indicator of egg freshness and used laboratory bench instrumentation [11,22].

In recent years, the availability of miniaturized and portable NIR instruments has provided the food industry with new opportunities for the use of this technology [23,24,25]. Miniaturized and portable NIR spectrometers have provided researchers with the ability to develop on-site and in-field applications of NIR spectroscopy [23,24,25]. Several fields have recognized the advantages of these instruments, including law enforcement and military applications, environmental analysis and food quality control and safety [23,24,25]. More importantly, these instruments provide the same accuracy and performance as the laboratory bench instruments [23,24,25]. Portable NIR devices are similar to laboratory benchtop instruments but are much smaller and lighter. This miniaturization has been made possible through the incorporation of Micro-Electro-Mechanical Systems (MEMS) or Micro-Opto-Electro-Mechanical optical systems (MOMS) [23,24,25]. This type of instrumentation has also become less expensive through the utilization of two main types of detectors, defined as an array or a single detector utilizing indium gallium arsenide (InGaAs) [23,24,25].

Current scenarios created by the coronavirus (COVID-19) pandemic, and ongoing conflicts in different parts of the world, have disrupted or slowed down many food chains. This has led to a surge in prices due to increased demand for food ingredients and commodities [5,26]. These issues have created an environment where fraud becomes prevalent, even in low-cost products such as eggs [5,26]. Consequently, assessing the authenticity and quality (e.g., freshness) of eggs at various stages of the supply chain (from farm to supermarket) using reliable, rapid, non-destructive, and cost-effective technologies has become paramount for the food manufacturing industry [5,27]. The use of NIR to authenticate the provenance of eggs laid by hens from different production systems has been shown to be a viable option [26,28,29]. It has been reported that a common fraudulent practice in the chicken egg supply chain is related to mislabeling the laying date to increase its expiration date (e.g., stale egg sold as fresh) [26,28].

In this study, the effectiveness of a portable near-infrared (NIR) instrument combined with chemometric techniques was assessed for monitoring and predicting the storage time of eggs under two storage conditions (room temperature and cold storage) collected from two production systems, namely cage and free-range.

## 2. Materials and Methods

Fresh eggs from two production systems, namely cage (C: *n* = 540) and free-range (FR: *n* = 540), were purchased from the same local farm (Brizy Farm Fresh Pty Lt, Gold Coast, Brisbane, QLD, Australia). All egg samples used were of the same white color, flawless and devoid of cracks and had been pre-washed with potable water, as is the practice in the industry. A total of 1080 eggs were randomly allocated into two storage conditions (N = 540, *n* = 270 per production system), namely cold storage (CT) (4 ± 0.5 °C) and room temperature (RT) (25 ± 2 °C) with relative humidity [40 to 70% relative humidity (RH)]. Data were recorded at 4, 16, 37, 51 and 58 days of storage following protocols defined by the industry and other authors [5,6,8]. For each combination of storage time conditions (CT and RT) and production systems (cage and free-range), 20 samples were randomly selected and analyzed from day 16 onwards at each data point (days of storage), weighed before the spectra were collected and analyzed using NIR. After the NIR analysis (spectra collection), all egg samples (type of storage and production systems) were broken and analyzed for freshness using the HU method, albumen, yolk color and albumen height [3,9]. The albumen height, Haugh units and yolk color were measured using the Egg Multi Tested apparatus (EMT-5200; Robotmation Co. Ltd., Tokyo, Japan) [3,9]. The yolk color scoring system used in the EMT-500 is based on the 1 to 15 scale of the DSM (previously Roche scale) yolk color fan scoring system [3,9]. The HU values are reported in this paper.

**Figure 1:**
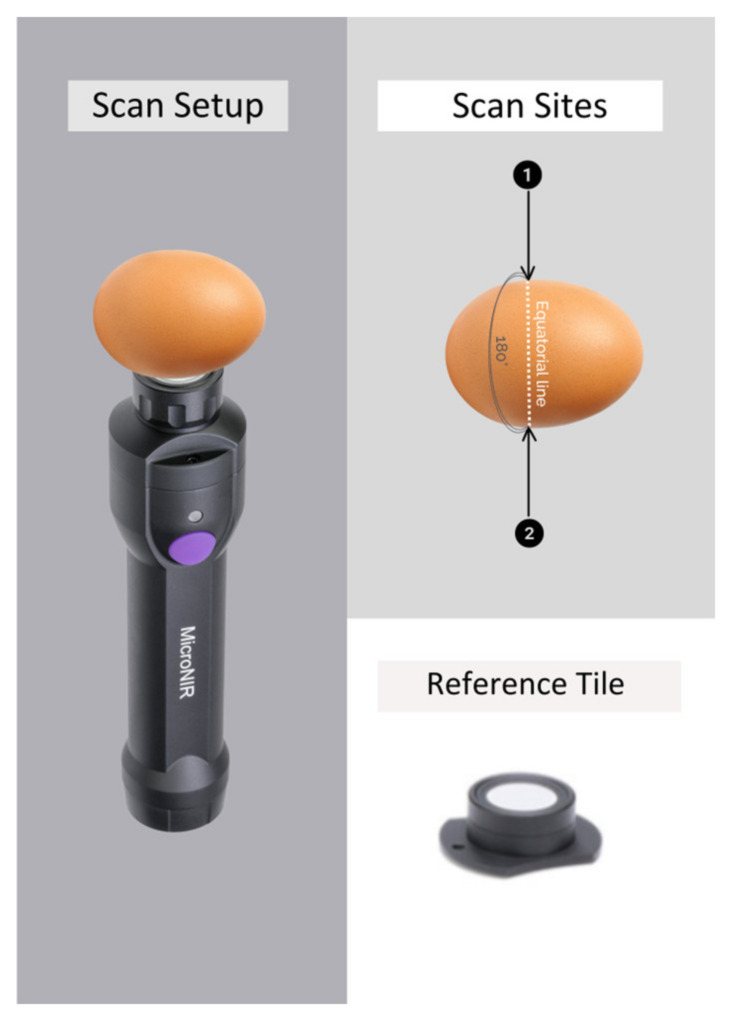
Set up, scan sites and reference tile (Spectralon^®^) used to collect the NIR spectra of the egg samples.

The NIR spectra of the intact egg samples were collected at two points of the equatorial line of the egg separated by 180°. This was achieved by using a portable NIR spectrophotometer (Micro-NIR 1700, Viavi, Milpitas, CA, USA) working in the 950–1600 nm wavelength range, with a spectral resolution of 10 nm with no moving parts (Viavi Solutions, 2015, Milipitas, CA, USA) [29]. The MicroNIR software was used to control the instrument and collect the spectra (MicroNIR Prov 3.1, Viavi, Milpitas, CA, USA). The parameters for spectral data acquisition were configured with an integration time of 50 ms and an average of 50 scans (MicroNIR Prov 3.1, Viavi, Milpitas, CA, USA). To calculate absorbance/reflectance, reference spectra of a Spectralon^®^ were collected every 20 samples [29]. Figure 1 shows the set-up utilized to collect the NIR spectra of the egg samples analyzed.

The NIR data were transformed with Savitzky–Golay second derivative (21 smoothing points and second polynomial order) before interpretation and chemometric analysis [30,31]. Principal component analysis and partial least-squares regression analysis (PLS) were used to analyze and interpret any trends in the data set and to develop models to predict storage time (The Unscrambler X, CAMO Analytics AS, Oslo, Norway). Full cross-validation (leave one out) was used to develop and validate both the PCA and PLS regression models [30,32,33,34]. The dataset was divided into calibration and validation sets using the Kennard–Stone algorithm [35]. A total of 700 uniformly distributed samples were selected, of which 490 were used to develop the calibration models and 210 were used for validation purposes. The Kennard–Stone algorithm allows data partitioning to be performed where knowledge of the training (calibration) dataset does not affect the test dataset (validation), and the predictive power of the created model is subsequently increased [35]. The models were evaluated using the coefficient of determination in cross-validation (R^2^_cv_), the standard error in cross-validation (SECV), standard error in prediction (SEP), bias and slope [30,32,33].

The impact of storage and production system on the cross-validated date for storage time was assessed. The resulting standard SECV of the model was compared using Fisher’s test (F value) [36,37]. The F value was calculated as F = SECV2/SECV1, where the relationship SECV1 < SECV2 was used [37]. The calculated F value was compared with the critical F value (F critical (1 − α, n1 − 1, n2 − 2)) obtained from the F distribution table. Here, α represents the significance level (α = 0.05 in this experiment), n1 the number of samples used in data set 1 and n2 the number of samples used in data set 2, (n1, n2, … = varies in each experiment). The differences in SECV were considered significant when F > F limit.

## 3. Results and Discussion

**Figure 2:**
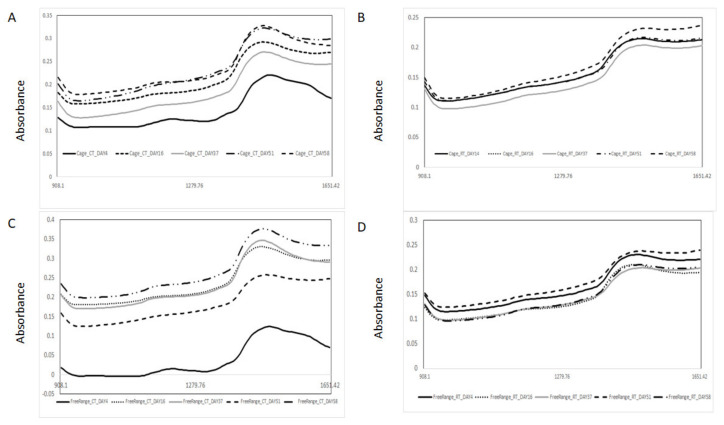
Raw mean near-infrared spectra of egg samples from cold (CT) and room (RT) temperature storage conditions and production systems (cage and free-range) analyzed using near-infrared spectroscopy. (**A**) cage and CT, (**B**) cage and RT, (**C**) free range and CT, (**D**) free range and RT.

**Figure 3:**
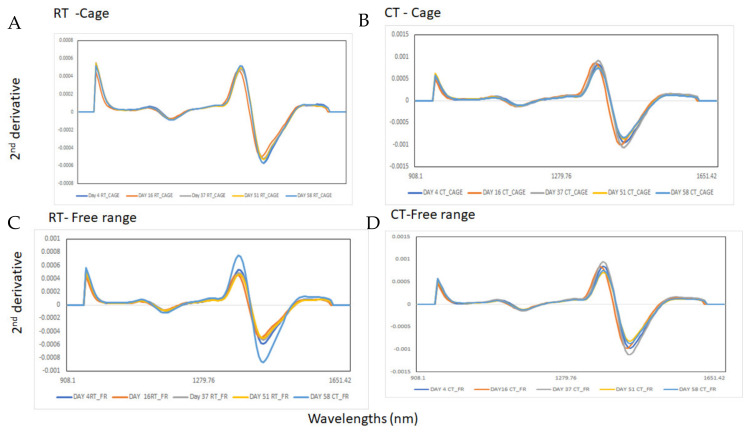
Second derivative mean (21 smoothing points; second polynomial order) near-infrared spectra of egg samples from cold (CT) and room (RT) temperature storage conditions and production systems (cage and free-range) analyzed using near-infrared spectroscopy. (**A**) RT and cage, (**B**) CT and cage, (**C**) RT and free range and (**D**) CT and free-range.

Figure 2 shows the raw mean NIR spectra of the egg samples analyzed in each storage time condition and different production systems. Figure 3A–D shows the second derivative NIR mean spectra of samples stored at RT and CT (Panel A: RT and cage, Panel B: CT and cage, Panel C: RT and free range and Panel D: CT and free-range). Two main features were observed in the second derivative NIR spectra around 1170 nm and 1440 nm, associated with C–H and O–H stretching bonds, respectively [38,39,40]. The absorbance around 1170 nm (between 1160 to 1180 nm) might be associated with C–H and C–H_3_ bonds [38,39,40]. This region can be associated with the absorption of fatty acids containing cis double bonds (second overtones C–H) as reported by Sato and collaborators (1991) [29,41]. However, the main differences in the NIR spectra of the egg samples collected from the different days and storage treatments were observed around 1440 nm (O–H bonds). This wavelength may be associated with the loss of water through the shell during storage, as well as the moisture content within the egg, as reported by other authors [17,18]. Other authors also indicated that the absorbance around 1450 nm might be associated with the first overtone of O–H stretching bonds. An increase in the absorbance values at this wavelength might be directly related to changes in protein structure during egg storage [22,29]. A recent study by Chen and co-workers [42] stated that the wavelength region between 1400 and 1500 nm might also be associated with the presence of N–H secondary bonds corresponding to amine groups [42]. They further indicated that the NIR spectra of whole eggs are derived from the overlapping of signals of various egg constituents (e.g., protein, lipids) [29,42]. This overlapping contributes to the spectral differences observed among the different types of eggs (e.g., cage vs. free-range; storage temperature). However, it is important to note that most of these spectral characteristics can be considered as being less prominent [42]. In this study, subtle changes were observed around this wavelength. These were not only in the absorbance value itself but also a shift on the absorbance of this wavelength towards higher wavelengths. This shift was dependent on the storage conditions (CT vs. RT) and the production system (cage vs. free-range). However, no clear trend can be definitively linked to either number of storage days or storage conditions (CT vs. RT).

**Figure 4:**
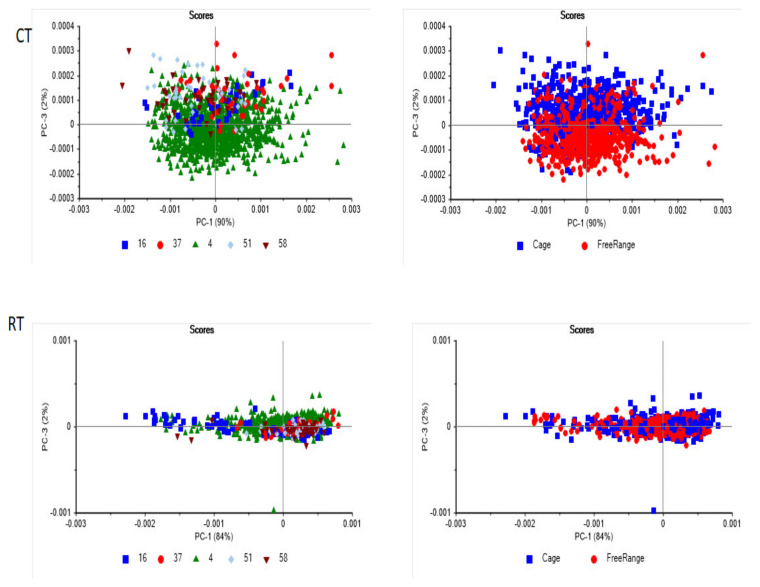
Principal component score plot of egg samples from cold (CT) (**Top**) and room temperature (RT) (**Bottom**) storage conditions and production systems (cage and free-range) analyzed using near-infrared spectroscopy.

The PCA scores plot of the whole egg samples from the storage conditions (CT and RT) are shown in Figure 4. The first three PCA components from the CT treatment explained 98% of the variance, while for egg samples from the RT treatment 97% of the variance was explained. In the case of egg samples from the CT treatment, plotting PC1 (90%) vs. PC3 (2%) shows a separation between cage and free-range egg samples. However, no trends or patterns were observed in the case of the eggs obtained from the RT treatment.

**Figure 5:**
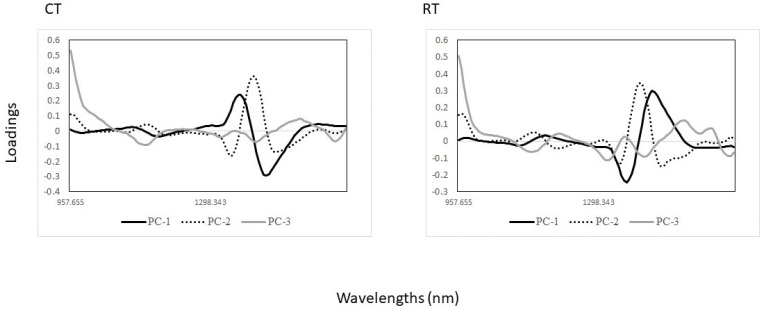
Loadings derived from the PCA analysis of egg samples from cold (CT) and room (RT) temperature storage conditions and production systems (cage and free-range) analyzed using near-infrared spectroscopy.

The PCA loadings were also different for each of the PCA models (Figure 5). The bands around 1193 nm (C–H and C–H_3_ bonds), 1397 nm (C–H combinations and C–H_2_ bonds) and 1508 nm (N–H) play a crucial role in explaining the differences observed in the PCA score plots [38,39,40]. The separation between the cage and free-range egg samples under CT storage was explained by wavelengths around 1140 nm (C–H and C–H_3_ bonds) which is associated with aromatic groups as well as fatty acids containing *cis* double bonds [41,43], around 1354 nm (C–H combination bonds) and around 1428 nm (O–H bonds) associated with moisture and proteins (Figure 5, CT) [38,39,40,43]. Although no clear separation between cage and free-range egg samples under RT storage was observed, the highest loadings were observed at similar wavelengths as reported in the previous section (Figure 5, RT).

**Figure 6:**
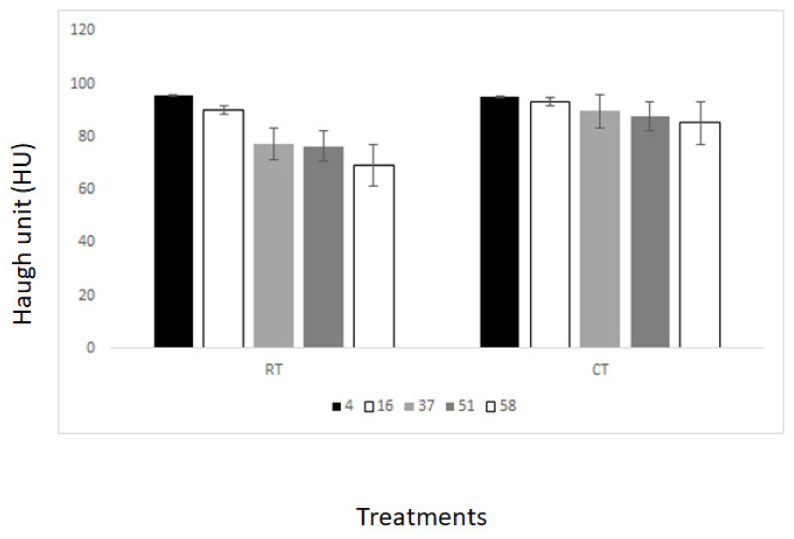
Changes in the Haugh units (HU) (freshness) in the egg samples from cold (CT) and room (RT) temperature storage conditions.

**Figure 7:**
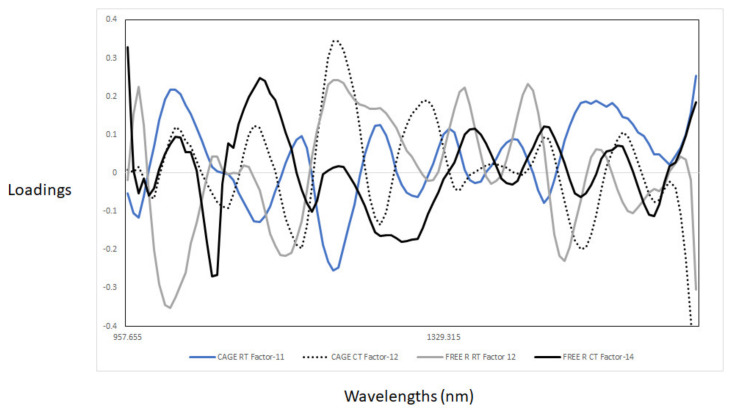
Optimal partial least-squares loadings were used during the development of the cross-validation models for the prediction of weeks of storage in the different combinations of storage conditions and production systems.

The freshness of the egg samples was assessed using the HU method. Figure 6 shows the changes in HU units in the egg samples analyzed from both CT and RT storage conditions, as well as from the cage and free-range production systems. The HU values varied depending on the storage conditions. For the egg samples from the RT storage condition, the HU values ranged from 95.8 to 69.4. On the other hand, the samples from the CT storage condition showed less variation, ranging from 95.3 to 85.2. Internal biochemical and chemical changes were attributed to explain the differences between production systems (e.g., changes in protein composition, water loss, changes in albumin and yolk color, etc.). These changes might contribute to explain the differences observed between cage and free-range egg samples. Some of these changes have been observed in the PLS loadings (Figure 7). Similar results were reported by others [43,44,45].

Table 1 presents the cross-validation statistics for predicting storage time for eggs from both cage and free-range production systems. The statistics varied depending on the combination of storage and production systems. The storage time of cage eggs under RT gave the poorest calibration results (R^2^_CV_: 0.67 and SECV: 7.64). The best R^2^_CV_ and SECV for the predicting storage days were obtained using egg samples from both cage and free-range production systems under CT storage. Specifically, the cage eggs had an R^2^_CV_ of 0.84 and an SECV of 5.38, while the free-range eggs had an R^2^_CV_ of 0.83 and an SECV of 5.52. In addition, free-range egg samples from the RT treatment also showed good results with an R^2^_CV_ of 0.82 and an SECV of 5.61. The standard error in prediction (SEP) values were 7.96, 5.56, 5.8 and 5.4 for cage RT, cage CT, free-range RT and free-range CT, respectively. Overall, the SECV obtained for predicting the storage days of cage eggs under RT was statistically significant different from the other three (*p* < 0.05). The RPD values obtained were 1.8, 3.2, 3.0 and 3.2 for cage RT, free-range RT, cage CT and free-range CT, respectively. The RPD values showed that the cross-validation models were adequate to classify samples according to days of storage, except for the egg samples from cage and RT storage conditions.

The PLS regression loadings used to predict the storage days varied between the models (Figure 7). The pattern in the loadings for caged eggs and RT treatment was different, while for CT cage and free-range eggs, as well as RT free-range eggs, they were similar. The highest loadings for the calibration models using the cage eggs and RT treatment were observed around 1106 nm and 1199 nm, associated with C–H aromatic groups, C–H and C–H_3_ bonds; around 1298 nm (C–H combinations); and around 1445 nm (O–H) associated with moisture and protein content groups [29,42,43]. The highest loadings for the other three models were observed in the regions between 1140 and 1190 nm (C–H aromatic groups, C–H and C–H_3_ bonds), between 1450 and 1490 nm (O–H) and around 1570 nm. In addition, a loading around 1027 nm (N–H aromatic group) [29,40] was observed for the free-range samples from the RT treatment. These results can be attributed to the effects of cold storage. It has been reported that free-range eggs tend to maintain their freshness better compared to cage eggs [44,45,46]. The classification results obtained in this study were better than those reported by Zhao et al. [7] and Fu et al. [10] using PLS regression. However, the results were similar to those reported by Lin et al. [20] using a bench instrument and Cruz-Tirado et al. [39] using a portable NIR spectrophotometer.

These results suggest that a portable NIR instrument could be viable as a rapid tool for predicting egg freshness in a non-targeted approach. The use of NIR spectroscopy could provide a rapid and efficient method for evaluating the freshness of eggs in the supply chain. This technique can also serve as a valuable decision-making tool for the egg industry, allowing for effective monitoring of storage times and determining the optimal time for selling.

## 4. Conclusions

This study demonstrated that NIR spectroscopy can non-destructively predict the storage time of intact egg samples from two production systems (cage and free-range) under both cold and room temperature (CT and RT) storage conditions. The best cross-validation and prediction statistics were obtained from free-range samples under CT storage conditions. The interpretation of the loadings also indicated that different spectral information was used by the PLS models. The method evaluated in this study is simple, rapid (±7 s per scan), environmentally friendly and avoids destroying the sample. The use of handheld NIR devices is expected to become an alternative to other routine methods used by the industry to assess egg freshness. Though the results of the present study are promising, further research is still needed to validate the existing PLS models using independent sets of samples that present a broader data set and that take into account the factors that could influence the chemical composition of the eggs (produced in different production systems, from layers fed different diets, etc.) and eggs stored under different conditions, as well as to explore the potential of this technique to predict additional egg quality parameters associated with freshness.

## Figures and Tables

**Table 1:** Cross-validation and prediction statistics for the measurement of days of storage in eggs from cage and free-range hens analyzed using near-infrared spectroscopy.

	Cage		Free-Range	
	RT	CT	RT	CT
R^2^_CV_	0.67	0.84	0.82	0.83
SECV (days)	7.64 ^a^	5.38 ^b^	5.61 ^b^	5.32 ^b^
bias	−0.01	0.13	0.01	0.12
slope	0.71	0.85	0.84	0.86
SEP	7.96	5.56	5.80	5.42
LV	11	12	12	14
RPD	1.8	3.0	3.2	3.2

CT: cold temperature; RT: room temperature; R^2^_CV_: coefficient of determination in cross validation, LV: latent variables, SECV: coefficient of cross validation, SEP: standard error of prediction, RPD: residual predictive deviation. Different letters in the same row denote statistically significant differences (*p* < 0.05).

## Data Availability

Data is contained within the article.
